# Prediction of *N*-Methyl-D-Aspartate Receptor GluN1-Ligand Binding Affinity by a Novel SVM-Pose/SVM-Score Combinatorial Ensemble Docking Scheme

**DOI:** 10.1038/srep40053

**Published:** 2017-01-06

**Authors:** Max K. Leong, Ren-Guei Syu, Yi-Lung Ding, Ching-Feng Weng

**Affiliations:** 1 Department of Chemistry, National Dong Hwa University, Shoufeng, Hualien 97401, Taiwan; 2 Department of Life Science and Institute of Biotechnology, National Dong Hwa University, Shoufeng, Hualien 97401, Taiwan

## Abstract

The glycine-binding site of the *N*-methyl-D-aspartate receptor (NMDAR) subunit GluN1 is a potential pharmacological target for neurodegenerative disorders. A novel combinatorial ensemble docking scheme using ligand and protein conformation ensembles and customized support vector machine (SVM)-based models to select the docked pose and to predict the docking score was generated for predicting the NMDAR GluN1-ligand binding affinity. The predicted root mean square deviation (RMSD) values in pose by SVM-Pose models were found to be in good agreement with the observed values (*n* = 30, *r*^2^ = 0.928–0.988, 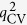 = 0.894–0.954, RMSE = 0.002–0.412, *s* = 0.001–0.214), and the predicted p*K*_i_ values by SVM-Score were found to be in good agreement with the observed values for the training samples (*n* = 24, *r*^2^ = 0.967, 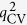 = 0.899, RMSE = 0.295, *s* = 0.170) and test samples (*n* = 13, *q*^2^ = 0.894, RMSE = 0.437, *s* = 0.202). When subjected to various statistical validations, the developed SVM-Pose and SVM-Score models consistently met the most stringent criteria. A mock test asserted the predictivity of this novel docking scheme. Collectively, this accurate novel combinatorial ensemble docking scheme can be used to predict the NMDAR GluN1-ligand binding affinity for facilitating drug discovery.

*N*-methyl-D-aspartate receptors (NMDARs), which are family members of ionotropic glutamate receptors (iGluRs), are expressed in the central nervous system (CNS) and play critical roles in a variety of physiological processes, such as neuronal development, synaptic plasticity, learning, memory, and motor function^1^,^2^. Moreover, it has been reported that NMDARs are profoundly implicated in various neurodegenerative disorders, such as Parkinson’s disease (PD), Alzheimer’s disease (AD), Schizophrenia, pain, and depression, and have been proposed as putative therapeutic targets in treating neurodegenerative illness^3^.

NMDARs are heteromeric assemblies of GluN1, GluN2, and GluN3 subunits, which were previously named as NR1, NR2, and NR3, respectively^4^. Four GluN2 isoforms (GluN2A-D) and two GluN3 isoforms (GluN3A and GluN3B) have also been identified. NMDARs form tetrameric complexes *in vivo* that consist of two GluN1 subunits and two GluN2 subunits or two GluN1 subunits and two GluN3 subunits^5^. Different subunits and, consequently, different subunit compositions have distinct biophysical, pharmacological, and signaling properties^6^.

In addition to therapeutic agents that can interact with NMDARs at the glycine and glutamate binding sites, channel blockers and positive allosteric modulators (PAMs) or negative allosteric modulators (NAMs)^7^,^8^ can also modulate NMDAR activity. The complexity in subunit combinations leads to diverse physiological functions as well as their roles in neurological diseases^9^. For instance, the binding affinity of NMDA antagonist ifenprodil at GluN1/GluN2A is about 400-fold lower than at GluN1/GluN2B^10^. Of various subunits and their combinations, glycine can bind to GluN1 and GluN3 subunits^11^,^12^ with pharmacological and structural differences in both binding sites^13^,^14^. More importantly, it has been suggested that the glycine binding site of GluN1 is a potential pharmacological target for treating PD, schizophrenia, traumatic brain injury, and anxiety^15^,^16^,^17^,^18^.

Numerous docking studies have been previously performed based on a single (crystal or homology) protein structure^19^,^20^,^21^,^22^,^23^,^24^,^25^,^26^. Nevertheless, NMDARs are highly flexible *per se* as illustrated by published crystal structures, namely GluN1 in co-complexes with glycine (PDB code: 1PB7), 5,7-dichlorokynurenic acid (DCKA) (PDB code: 1PBQ), and cycloleucine (PDB code: 1Y1M)^27^,^28^. When superimposed, these proteins show substantial structural discrepancies as displayed by Fig. 1, in which protein structures excerpted from co-complex structures were aligned, especially residues Thr^126^, Arg^131^, Ser^180^, and Asp^224^ that constitute the putative binding pocket and contribute to the plastic nature of GluN1. This is completely consistent with dynamic stimulations^29^,^30^. In addition to substantial conformation change, the promiscuous nature of the GluN1 glycine binding site can also be manifested by its substantial variations in the size of binding pocket bound with structurally distinct ligands. For example, the binding pocket volume of the glycine-bound GluN1 (PDB code: 1PB7) is about 93.26 Å^3^ as calculated by the *CASTp* package (available at http://sts-fw.bioengr.uic.edu/castp/calculation.php) using a 1.4 Å probe, whereas that of DCKA-bound GluN1 (PDB code: 1PBQ) is about 198.56 Å^3^, yielding a 112% increase in volume. More complexity can be added because GluN1 can undergo conformational change upon binding with a ligand^31^,^32^.

As such, the plastic nature of GluN1 cannot be fully addressed by a single GluN1 structure to accurately model the protein-ligand interaction except molecular dynamics that, in turn, will be less practically useful due to its low computational throughput^33^. Conversely, ensemble docking, which is carried out by placing a ligand into several target structures and selecting the best fit pose by score or root mean square deviation (RMSD) values if applicable^34^, seems to be a plausible alternative since it has been demonstrated that ensemble docking performs better than docking with a single protein structure^35^.

Most docking calculations are carried out using a single ligand conformation, despite that ligands can be flexibly docked. However, it has been demonstrated that the ligand initial conformation plays a significant role in docking accuracy, suggesting that it is necessary to search for a number of stable ligand conformations prior to docking^36^. In other words, the ligand ensemble docking approach should be adopted by which an ensemble of ligand conformations is generated and then each conformer is docked to the binding pocket^37^.

The combination of ligand and protein conformations results into a combinatorial ensemble docking that can yield a great number of poses, leading to a serious challenge to select the best fit pose. Since, normally, (ensemble) docking relies on a single scoring function to select or to rank the best pose that shows the lowest RMSD from the bound ligand structure, *viz*. the native binding pose, among all produced docked poses. Nonetheless, even an accurate scoring function cannot satisfactorily select or rank the best pose^38^. It can be attributed to the fact that RMSDs in pose do not always well correlate with scores given by a scoring function^39^,^40^,^41^,^42^.

As such, substantial efforts have been devoted to the development of novel schemes to select the best docked pose. For instance, it has been proposed to select the best pose by the consensus scoring scheme (*vide infra*) instead of a single scoring function^43^. In fact, it has been demonstrated by numerous studies that consensus scoring schemes could perform better than single scoring functions^44^,^45^. Of various versions of consensus scoring^46^, the most prevalent ones are rank-by-number, rank-by-rank, and rank-by-vote^47^ by which the docked poses are re-ranked by averaging (or summing) the scores with a panel of scoring functions, by the averaged rankings based on various scoring functions, and by the final scores based on ranking votes gathered from various scoring functions, respectively^40^,^48^,^49^.

Nevertheless, there are a number of critical issues associated with consensus scoring, namely different numerical spans and units given by various scoring functions, different genres of scoring functions (*vide infra*), and linear combinations of consensus scoring functions^47^. Those problems seemingly can be resolved by machine learning (ML) schemes, which can establish a nonlinear relationship between input and output variables. In fact, it has been demonstrated that a scoring function developed by an artificial neural network (ANN) performed better than the conventional linear consensus scoring functions^50^. Of various ML schemes^51^, support vector machine (SVM), which was invented by Vapnik *et al*. in 1995^52^ and has been extensively applied to a broad range of studies^53^,^54^,^55^, performs better than any other ML techniques, such as ANN, genetic algorithm (GA), and random forest (RF) as demonstrated by empirical studies^56^,^57^, suggesting that an SVM-based model can actually perform better than any other ML-based schemes in selecting/ranking docked poses.

The selected docked poses are subjected to further evaluations by a scoring function, which is a mathematical model to produce scores that represent the ligand-protein binding affinities and detailed description of scoring function can be explained elsewhere^58^. Scoring functions can be basically categorized into knowledge-based, empirical, and force field-based types^59^. Force field-based scoring functions are parameterized based on the potential energy functions and parameters deduced from quantum mechanical calculations and experimental data. The binding free energy in the empirical scoring function is calculated by summing all contributions from various empirical energy terms with different weights to linearly fit the binding affinities of a set of protein-ligand complexes. Knowledge-based scoring functions are based on atomic interaction free energy parameters derived from the observed frequencies of interacting atom-atom contacts in protein-ligand complexes via a procedure based on statistical mechanics.

It is normally assumed that scores produced by scoring functions are linearly correlated with the experimentally determined binding affinities of the protein–ligand complexes of known 3D structures. However, such assumption is not always true^59^. Consensus scoring proposed by Charifson *et al*. was purported to remedy such problems by linearly combining scoring functions to predict the ligand-protein binding affinity^60^. In fact, it has been demonstrated that consensus scoring functions indeed perform better than single scoring functions^61^,^62^. Conversely, it is exceptionally difficult, if not completely impossible, to observe a linear relationship between binding affinities and scores yielded by scoring functions or consensus scoring functions, even though assorted variations, including combinations of different classes of scoring functions, have been proposed. This is mainly due to the nonlinear additive nature of the non-covalent interactions used to construct a scoring function^63^ as demonstrated by the fact that the correlation coefficients between scores and binding affinities are often no more than 0.5^42^.

As such, nonlinear approaches such as ML-based scheme seem to be better alternatives as compared with their linear counterparts^64^. For example, ANN, RF, and SVM have been adopted by Betzi *et al*.^50^, Li *et al*.^65^, and Zilian and Sotriffer^66^ to develop *GFscore, ID-Score*, and *SFCscore*^*RF*^, respectively; which unequivocally performed better than single and consensus scoring functions. In addition, it has been demonstrated that a customized SVM scoring function for a specific target can even execute better^63^,^67^,^68^.

Accordingly, it is plausibly to expect that a docking study, in which the docked poses and docking scores are selected and calculated by customized SVM models for a specific target, should perform extremely well. In addition, it is generally believed that the more training samples, the better a predictive model. As such, better customized SVM-Pose and SVM-Score models can be yielded once there are more ligand-protein co-complex structures with the corresponding binding affinities that, in turn, will require ensemble docking^69^. The objective of this study was to accurately model the ligand binding to the NMDAR subunit GluN1 by this novel SVM-Pose/SVM-Score combinatorial ensemble docking scheme to facilitate drug discovery to find novel therapeutics for the potential treatment of neurological disorder.

## Materials and Methods

### Protein preparation

Of published NMDA GluN1 structures^27^,^28^,^70^,^71^,^72^,^73^,^74^, seven protein structures with PDB codes 1PB7, 1PB8, 1PB9, 1PBQ, 1Y1M, 1Y1Z, and 1Y20 (co-complexes with, respectively, glycine, D-serine, D-cycloserine, DCKA, cyclo-leucine, 1-aminocyclobutane-1-carboxylic acid (ACBC), and 1-aminocyclopropane-1-carboxylic acid (ACPC)) were adopted because of their consistency with the assay system to determine *K*_i_ values that is of critical importance to scoring function development (*vide infra*).

Initially, water molecules were removed and hydrogen atoms were added using the *Macromolecule* preparation protocol in *Discovery Studio* (Accelrys, San Diego, CA). Each protein structure was subjected to energy relaxation to remove the clashes among atoms^75^ using the steepest descent (SD) method with the selection of AMBER force field^76^ until the gradient was smaller than 0.3 with respect to the previous optimization step. The binding pocket residues of every protein structure were initially searched by *LigPlot*^77^ and the volume of binding pocket was then computed by *CASTp* using the key residuals, namely Phe^92^, Pro^124^, Leu^125^, Thr^126^, Arg^131^, Ser^179^, Ser^180^, Val^181^, Trp^223^, Asp^224^, and Phe^250^.

### Ligand preparation

To construct a non-redundant ligand conformation ensemble, each ligand was subjected to conformational search to generate the low-energy conformations using mixed Monte Carlo multiple minimum (MCMM)^78^/low mode^79^ implemented in the *MacroModel* package (Schrödinger, Portland, OR). The energy minimization was carried out by the truncated-Newton conjugated gradient method (TNCG) with the selection of MMFFs force field^80^. The most stable 10 unique structures were selected for initial docking conformers.

### Ensemble docking

Docking calculations were carried out using the *GOLD* package (Cambridge Crystallographic Data Centre, Cambridge, UK) because of its excellent performance in the case of NMDA GluN1^81^. *GOLD* is a stochastic system based on GA to flexibly dock ligand into the binding pocket of target protein. The docked results are evaluated by a fitness function, which is comprised of van der Waals, hydrogen bond, and internal interactions. In each docking calculation, *Gold* performs a number of independent docking runs and generates numerous poses. Three poses were selected in each run by their fitness values. The ensemble docking was carried out by docking each ligand conformer into the selected crystal structures by *Gold* and each ligand was docked 10 times to possibly eliminate any random bias.

### SVM-Pose

**Figure 2:**
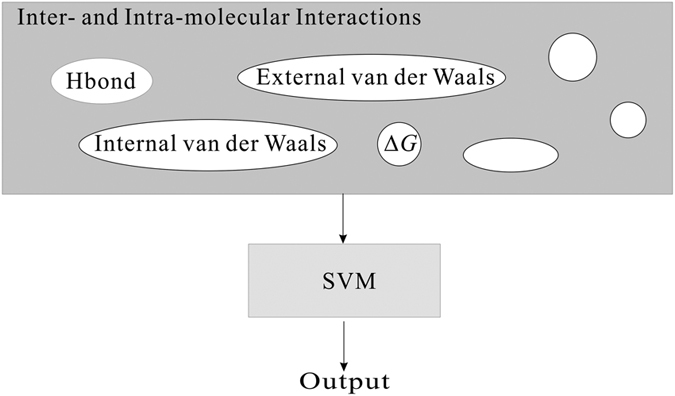
Schematic presentation of SVM-Pose architecture.

Figure 2 schematically represents the architecture of SVM-Pose. Each SVM-Pose model was derived from each crystal structure based on 30 docked poses, and seven customized SVM-Pose models were built. The inter- and intra-molecular interactions associated with the docked poses, which were evaluated by the scoring functions *GoldScore*^82^, *ChemScore*^83^, *LigScore1, LigScore2*^84^, *Piecewise Linear Potential 1* (*PLP1*), *PLP2*^85^, *Jain*^86^, *Potential of Mean Force* (*PMF*)^87^, and *PMF4*^88^, were treated as the independent variables, *viz*. the SVM input, and the corresponding RMSD values between docked and native binding poses were treated as the dependent variables, *viz*. the SVM output.

The model development and verification were carried out using the modules *svm-train* and *svm-predict* implemented in the SVM package *LIBSVM* (software available at http://www.csie.ntu.edu.tw/~cjlin/libsvm). The runtime parameters, namely regression modes *ε*-SVR and *ν*-SVR, the associated *ε* and *ν*, cost *C*, and the width of the radial basis function (RBF) kernel *γ*, were systemically scanned in a parallel fashion using an in-house Perl script.

### SVM-Score

**Figure 3:**
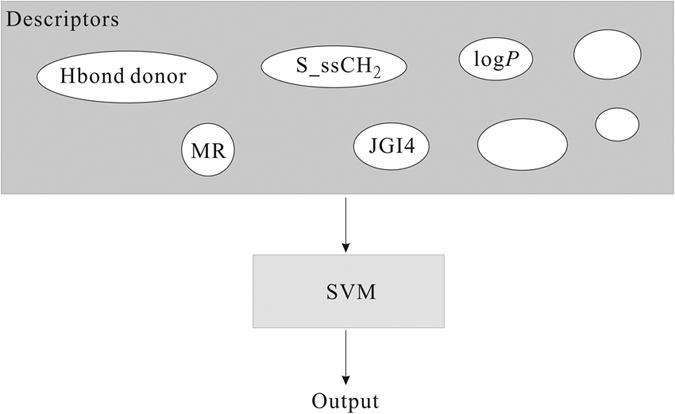
Schematic presentation of SVM-Score architecture.

The selected poses were further employed to develop SVM-Score, whose architecture is displayed in Fig. 3. There were only 7 bound ligands in the co-complex crystal structures available and their chemical structures are not dissimilar, which, in turn, will severely restrict the applicability of a developed model. Such limitations can be eased once more samples with more dissimilar structures are added to the collection and a better predictive model can be developed^89^. To further expand the training sample collection, a comprehensive literature search was carried out to retrieve the compounds, whose *K*_i_ values were also assayed by the same conditions for the bound ligands in the crystal structures. An additional 30 molecules were adopted after carefully scrutinizing the collected ligands to maintain structural clarity since compounds with different chirality can exert different binding affinities^90^.

The 30 unbound compounds were subjected to ensemble docking through the use of the same procedure previously described. Of all generated poses (7 protein structures × 10 ligand conformers × 3 produced poses from each docking calculation), only the one with the lowest predicted RMSD in pose was selected. The top-most docked poses for the 30 unbound and 7 bound molecules were divided into two groups, namely the training set and test set, to build the predictive model and to verify the developed model using the Kennard-Stone (KS) algorithm^91^ implemented in *MATLAB* (The Mathworks, Natick, MA) with a ca. 2:1 ratio. The *K*_i_ values of training samples spanned 7 orders of magnitude.

It has been shown that the adoption of more descriptors can significantly improve the performance of scoring functions in addition to protein-ligand empirical interactions^65^,^66^. Thus, *Discovery Studio* (Accelrys, San Diego, CA) and *E-Dragon* (available at the Website http://www.vcclab.org/lab/edragon/) were employed to generate more than 3000 more molecular descriptors. Descriptors were preprocessed by eliminating those missing for at least one compound or showing little or no discrimination against all training samples, followed by discarding those with intercorrelation values of *r*^2^ ≥ 0.64 to reduce the probability of spurious correlations^92^. In addition, descriptors were subjected to normalization by centering at the mean value and dividing by the standard deviation^93^.

The descriptor selection was initially carried out by genetic function algorithm (GFA) using the QSAR module of *Discovery Studio* because of its effectiveness and efficiency^94^. A further selection was executed by the recursive feature elimination (RFE)^95^ method to remove relatively unimportant descriptors. The selected descriptors, along with the intra- and inter-molecular interactions implemented in the scoring functions, were treated as the input of SVM-Score and the associated p*K*_i_ values served as output. The SVM calculations were carried out as previously mentioned.

### Predictive evaluation

The derived models were evaluated by the parameters, namely correlation coefficients *r*^2^ and *q*^2^ in the training set and external set, respectively. The correlation coefficient of 10-fold cross validation 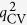 in the training set, the correlation coefficients 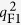, 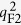, and 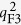
and concordance correlation coefficient (*CCC*) in the external set, various modified versions of *r*^2^, the residual Δ_*i*_, the root mean square error (RMSE), and the mean absolute error (MAE) (Table S1) for quantitative evaluation.

An *in silico* model can be considered as quantitatively predictive if it can meet the most stringent criteria proposed by Golbraikh *et al*.^96^, Ojha *et al*.^97^, Roy *et al*.^98^, and Chirico and Gramatica^99^,


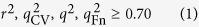



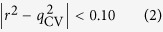



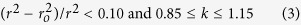



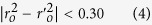



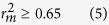



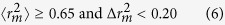



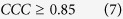


where *r* in equations (3),(4),(5),(6) represent the parameters *r* and *q* in the training set and external set, respectively.

Furthermore, the confusion matrix (Table S2) was constructed to calculate the Cooper statistics^100^, namely sensitivity, specificity, accuracy, and Matthews correlation coefficient, and Kubat’s G-mean^101^ (Table S3) to qualitatively assess a predictive model.

## Results

### Ensemble Docking

**Figure 4:**
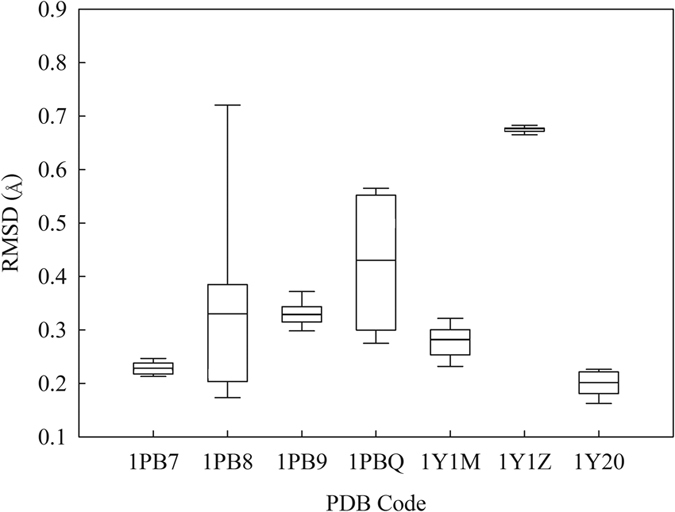
Box plot showing the RMSD values in pose using different native protein structures. Boxes represent the mean ± standard deviation, lines depict the median values, and whiskers denote the minimum and maximum values.

The docking calculations carried out by *Gold* are in excellent agreement with crystal structures as manifested by their small average RMSD values (Fig. 4), which displays the box plot of the RMSD minimum, maximum, mean, and standard deviation. For instance, the docking calculations based on the 5,7-DCKA bound crystal structure (PDB: chain B of 1PBQ) yielded RMSD values between 0.2 Å to 0.6 Å and an average RMSD of 0.43 Å for the 30 docked poses after 10 docking runs. Collectively, the average RMSD value of the 7 co-complex structures is 0.35 Å, which is much smaller than the threshold 2 Å as suggested^102^,^103^, indicating that *Gold* is suitable for this investigation since the bound ligand structures are highly reproducible by *Gold*.

### SVM-Pose

Seven SVM-Pose models, denoted by SVM-Pose^1PB7^, SVM-Pose^1PB8^, SVM-Pose^1PB9^, SVM-Pose^1PBQ^, SVM-Pose^1Y1M^, SVM-Pose^1Y1Z^, and SVM-Pose^1Y20^, were developed for the co-complex protein structures (PDB codes: 1PB7, 1PB8, 1PB9, 1PBQ, 1Y1M, 1Y1Z, 1Y20). Tables S4 and S5 list the optimal runtime conditions as well as the selected intra- and inter-molecular interactions excerpted from various scoring functions, respectively. Table S6 lists the predicted RMSD values in pose by 7 SVM-Pose models. Table 1 summarizes their associated statistical evaluations and validation requirements.

**Figure 5:**
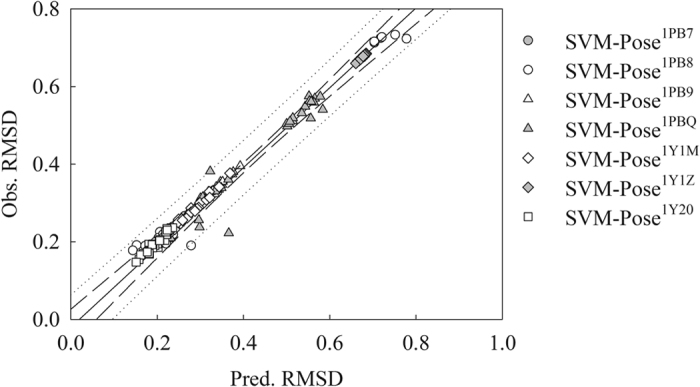
Observed RMSD versus the RMSD predicted by 7 SVM-Pose models, namely SVM-Pose^1PB7^ (gray circle), SVM-Pose^1PB8^ (open circle), SVM-Pose^1PB9^ (open triangle), SVM-Pose^1PBQ^ (gray triangle), SVM-Pose^1Y1M^ (open diamond), SVM-Pose^1Y1Z^ (gray diamond), SVM-Pose^1Y20^ (open square) and the ideal regression line.

The predicted RMSD values by the seven SVM-Pose models are in excellent agreement with observed values when applied to the protein structures that the predictive models were developed. Figure 5 displays the scatter plot of observed *vs*. predicted RMSD values by SVM-Pose models. For instance, they produced *r*^2^ values of more than 0.90 and *s* values of less than 0.25 (Table 1). In addition, they yielded almost negligible differences between *r*^2^ and 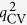 (no more than 0.10), suggesting that they were not statistically over-trained *per se*^104^.

The predictivity of generated SVM-Pose models were further assessed by the validation requirements proposed by Golbraikh *et al*.^96^, Ojha *et al*.^97^, Roy *et al*.^98^, and Chirico and Gramatica^99^. It can be found from Table 1, which summarizes the validation results, that the SVM-Pose models not only produced significant statistical values but also fulfilled all validation requirements. For instance, SVM-Pose^1PBQ^ produced an 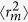 value of 0.86 and a 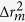 value of 0.07. Thus, it can be concluded that these theoretical models are highly accurate and predictive.

**Figure 6:**
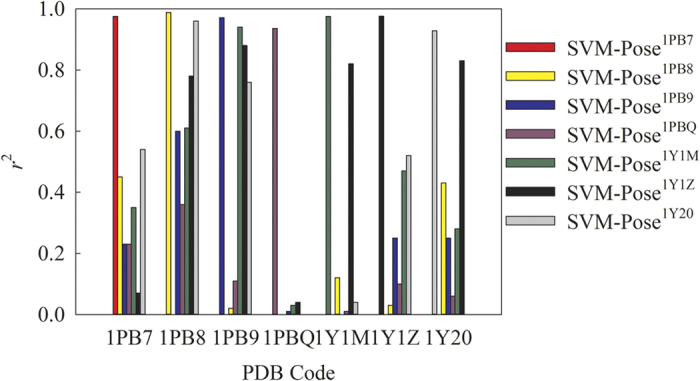
The correlation coefficient (*r*^2^) between predicted and observed RMSD in pose by 7 SVM-Pose models in 7 co-complex structures.

Nevertheless, the seven SVM-Pose models unequivocally showed deteriorated performances once applied to the other protein structures from which the SVM-Pose models were not derived, *viz*. non-native structures. Figure 6 displays the *r*^2^ values between predicted *vs.* observed RMSD values in pose by the 7 SVM-Pose models when applied to the 7 co-complex structures. For instance, SVM-Pose^1PB8^ developed from the co-complex structure with the PDB code 1PB8 gave rise to the highest *r*^2^ value when applied to its native protein structure with an *r*^2^ value of 0.99. Conversely, it gave rise to the *r*^2^ values of no more than 0.50 once it was applied to the other protein structures. The substantial performance discrepancies by the seven SVM-Pose models suggest that no single SVM-Pose model can consistently perform well for all of 7 protein structures. It is necessary to develop a customized model to predict RMSDs in pose for each individual protein conformation. Consequently, it is plausible to expect that poor pose selections can be yielded in the ensemble docking if the pose selection only relies on a single model.

### SVM-Score

Of 7 bound ligands and 30 unbound ligands included in the SVM-Score development, 24 and 13 molecules were randomly assigned to the training set and test set, respectively. Figure S1 shows the projection of all molecules enrolled in this investigation in chemical space, spanned by the first three principal components (PCs), explaining 93.9% of the variance in the original data. As displayed, both data sets exhibited high levels of similarity in the chemical space, whereas the bound ligands are positioned themselves far away from the unbound ligands, suggesting the high levels of dissimilarity between bound and unbound ligands that, in turn, can substantially augment the applicability domain (AD) of the derived scoring function. In addition, the high levels of biological and chemical similarity between both data sets can also be illustrated by Fig. S2, which displays the histograms of p*K*_i_, molecular weight (MW), surface area, and molecular volume (*V*_m_) in density form for all molecules in the training set and test set, suggesting the unbiased partition of data samples^105^.

**Figure 7:**
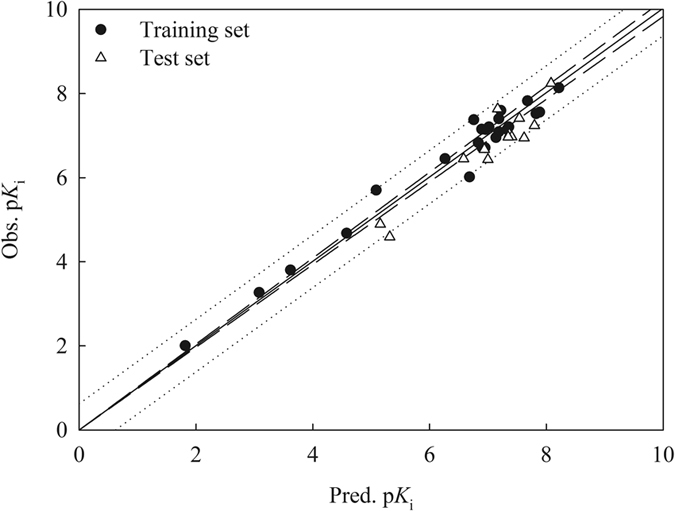
Observed p*K*_i_ *vs*. the p*K*_i_ predicted by SVM-Score for the molecules in the training set (solid circle) and test set (open triangle).

Table S7 lists the predicted p*K*_i_ values by SVM-Score and Table S4 shows the optimal runtime parameters. It can be observed that the predictions by SVM-Score are in good agreement with observed values for the molecules in the training set and test set as illustrated by Fig. 7, which displays the scatter plot of observed *vs*. predicted p*K*_*i*_ values in both data sets. Table 2 summarizes the statistical evaluations of SVM-Score. It can be found that SVM-Score produced insignificant prediction errors, suggesting that SVM-Score is an acute predictive model. For instance, the *s* values were only 0.170 and 0.202 in the training set and test set, respectively. Furthermore, SVM-Score gave rise to the high *r*^2^, *q*^2^, and 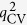 values of 0.967, 0.894, and 0.899, respectively, suggesting that SVM-Score is highly predictive. The negligible differences between *r*^2^ and *q*^2^ (0.073) and between *r*^2^ and 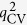 (0.068) unequivocally affirm that SVM was a well-trained model since it will produce at least one substantial difference in cases of overtraining. When subjected to the validation criteria proposed by Golbraikh *et al*.^96^, Ojha *et al*.^97^, Roy *et al*.^98^, and Chirico and Gramatica^99^ (equations (1),(2),(3),(4),(5),(6),(7)) to gauge the predictivity of a theoretical model, SVM-Score completely fulfilled all statistical validation requirements, indicating its high level of predictivity.

Table 3 lists all of interactions and descriptors selected to develop SVM-Score. In addition to the inter- and intra-molecular interactions excerpted from *ChemScore*, a number of descriptors were purported to augment the protein-ligand interactions. For instance, it has been found by Furukawa and Gouaux that hydrogen bond interactions play an important role in NMDA-ligand interactions^27^. The descriptor number of hydrogen-bond donor (HBD) was selected because of an *r* value of 0.782 between HBD and *Chemscore.Hbond* (Table S8). More importantly, scoring functions with the selection of HBD performed better than those with the selection of *Chemscore.Hbond* (data not shown).

Conversely, it seems unusual that the number of hydrogen-bond acceptor (HBA) was not selected since HBD and HBA play a significant role in NMDA-ligand interaction^106^. It can be observed that the p*K*_i_ values increased with increasing HBA as illustrated by Fig. S3. The absence of HBA can be attributed to the selected descriptor Atype_N_75, which describes specific types of nitrogen atom. It correlated with HBA well with an *r* value of 0.881 for the bound ligands. Conversely, this dependency was not observed for the unbound ligands (Table S8). As such, it is plausible to replace HBA by Atype_N_75 since the developed scoring functions with the selection of Atype_N_75 executed better than those with the selection of HBA (data not shown).

In addition, it has been found by Di Fabio *et al*. that the descriptor MR_omp_, which describes the total molar refractivity of substituents at *ortho, meta*, and *para* positions, was closely related to the NMDA-ligand binding affinity at the glycine binding site^107^. The adopted descriptor CIC1 was strongly correlated with MR with an *r* value of 0.800 (Table S8), suggesting that it is plausible to replace MR by CIC1 to describe such protein-ligand interaction.

It is of interest to observe that the dependence of the descriptor CC, which counts the number of chiral centers within a molecule, can be varied by chemotypes. More specifically, the bound ligands barely showed any relationship between CC and p*K*_i_ as manifested by its almost negligible *r* value (0.056), whereas the CC values of the unbound ligands were inversely correlated with p*K*_i_ (−0.622) (Table S8), suggesting that both types of ligands interact with proteins differently.

A number of Dragon descriptors were selected in this study. It is normally not straightforward to interpret Dragon descriptors. Nevertheless, it can be empirically observed that the descriptor JGI4, which is a topological charge index to measure the charge transfers between atom pairs^108^, was highly correlated to the p*K*_*i*_ values for the molecules with the acetylenic aromatic moiety with an *r* value of 0.794. This was merely 0.264 for the others (Table S8), suggesting that the molecular charge distribution plays a profound role in the NMDA-acetylenic aromatic interactions^109^. Additionally, descriptors S_ssCH_2_ and HATS6u were highly associated with p*K*_i_ with *r* values of −0.786 and −0.797, respectively, for the unbound ligands. They were −0.071 and −0.294, respectively, for the others (Table S8), suggesting that it is of necessity to adopt both descriptors to augment the protein-ligand interactions for the unbound ligands.

The selection of Atype_N_75, CC, JGI4, S_ssCH_2_, and HATS6u to render the interactions between protein and specific types of ligands manifests that nonlinear ML-based models can perform better than their linear counterparts and customized models, in turn, can execute better than their general counterparts. As such, it is plausible to expect that a customized SVM model should deliver outstanding performance in predicting binding affinity.

### Mock test

The developed SVM-Pose/SVM-Score combinatorial ensemble docking scheme was further subjected to test by the 10 quinoxalinones and quinazolinones assayed by McQuaid *et al*.^110^ to mimic real-world challenges. Nevertheless, these molecules were measured by the radioligand [^3^H]glycine, whereas all of molecules enrolled in this study were assayed using the radioligand [^3^H]MDL 105,519. The discrepancy in both systems actually does not pose an unsurmounted barrier since it has been reported by Baron *et al*. that the p*K*_i_ values obtained from both systems were highly correlated with an *r* of 0.90^111^. Thus, it is plausible to examine the SVM-Pose/SVM-Score combinatorial ensemble docking scheme with the molecules assayed by McQuaid *et al*. without significant errors.

**Figure 8:**
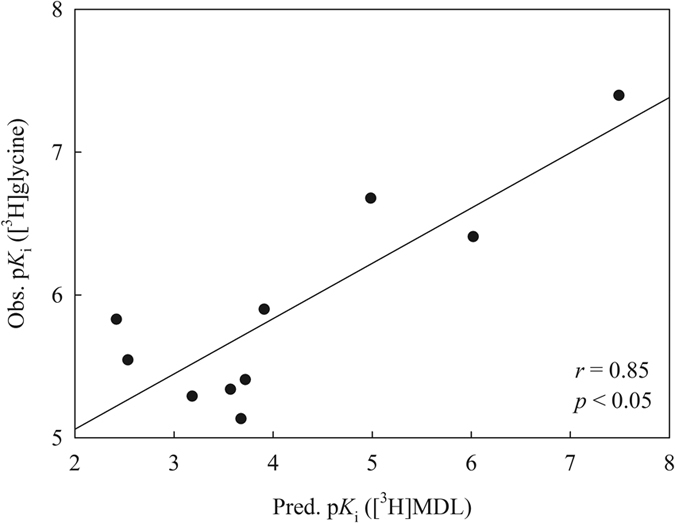
The observed p*K*_i_ values ([^3^H]glycine) *vs*. the predicted p*K*_i_ values ([^3^H]MDL) by SVM-Pose/SVM-Score ensemble docking.

Table S9 lists the tested results with the 10 molecules and Fig. 8 illustrates the obtained scatter plot. It can be observed that both systems were highly correlated with each other with an *r* of 0.85. The negligible difference between both parameters (0.90 *vs*. 0.85) suggests that the predictions by the SVM-Pose/SVM-Score combinatorial ensemble docking scheme can almost reproduce the experimental observations. Thus, this mock test unambiguously affirmed the predictivity of SVM-Pose/SVM-Score combinatorial ensemble docking.

## Discussion

**Figure 9:**
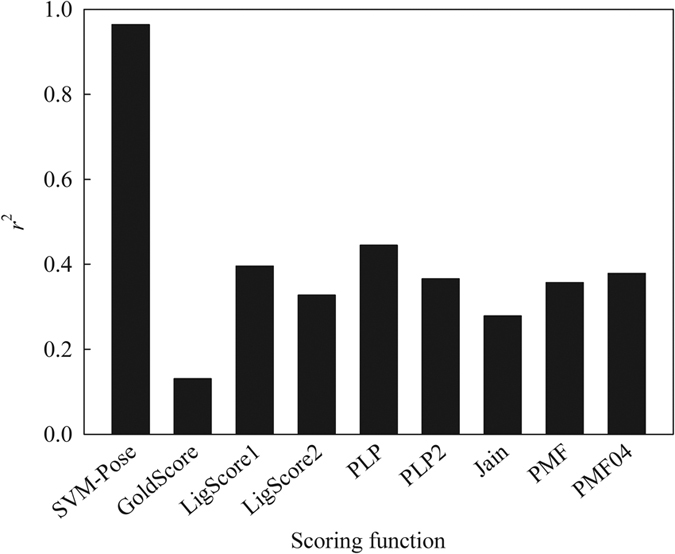
The correlation coefficient (*r*^2^) between predicted and observed RMSD by average SVM-Pose and various scoring functions.

It is well-established that pose and scoring play a pivotal role in docking^59^. Most docking studies rely only on a single scoring function to select top docked poses^40^. The knowledge-based scoring functions *PMF* and *PMF04*, the empirical scoring functions *PLP, PLP2, LigScore1*, and *LigScore2*, and the force-field scoring function *GoldScore* produced the *r*^2^ values of no more than 0.45 between calculated scores and RMSD values when applied to the 7 co-complex structures. Figure 9 displays the *r*^2^ values between RMSD values and scores evaluated by average SVM-Pose and various scoring functions, depicting the poor relationship between both parameters. This can lead to serious problems for pose selection since the scoring functions cannot always give the high scores to the poses with low RMSD values and such inconsistencies, in fact, are not uncommon^47^,^112^.

Conversely, the average SVM-Pose produced an *r*^2^ of 0.90 despite the SVM-Pose models were derived based on the intra- and inter-molecular interactions excerpted from the scoring functions (Table S5). This suggests that the nonlinear relationships between intra-molecular interactions and docked poses as well as between inter-molecular interactions and docked poses. In fact, this is completely consistent with observations made by Feher and Williams^113^. The superior performance of SMV-Pose models can be plausibly attributed to their outstanding capacity in non-linear regression when compared with the linear counterparts.

**Figure 10:**
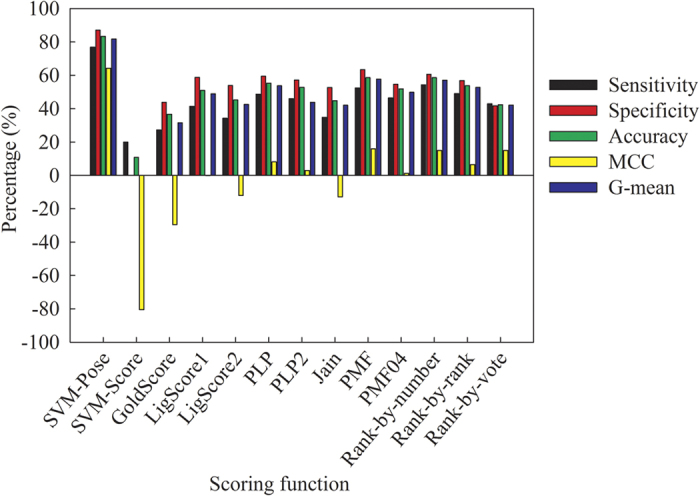
Sensitivity, specificity, accuracy, MCC, and G-mean evaluated by average SVM-Pose, SVM-Score, various scoring functions, and various consensus scoring schemes.

It can be argued that the major issue of pose selection is to accurately choose the docked pose with the lowest RMSD, *viz*. the top-most pose^41^. In other words, qualitative selection is more important than quantitative predictions of RMSD values. As such, it is of interest to evaluate the qualitative performances of SVM-Pose models and the scoring functions in the top-most pose selections using the Cooper statistics and Kubat’s G-mean (Table S3). Figure 10 presents the results. It can be observed that average SVM-Pose unequivocally performed better than the scoring functions in selecting the top-most poses. Of various scoring functions, *PMF* yielded the highest sensitivity, specificity, accuracy, and G-mean of ca. 60%, which are much smaller than those produced by average SVM-Pose (ca. 80%). Significant performance discrepancies between SVM-Pose models and the scoring functions occurred because there were only small variations in RMSD among docked poses (Fig. 4). As such, only customized ML-based SVM-Pose models can be sensitive enough to discriminate the top-most pose from the others when compared with their linear counterparts.

It has been demonstrated that consensus scoring schemes performed better than single scoring functions in selecting the top-most poses^44^,^45^. Accordingly, it is of interest to compare the performance of average SVM-Pose with various consensus scoring schemes, namely rank-by-number, rank-by-rank, and rank-by-vote. Figure 10 displays the comparison results. It can be observed that the rank-by-number scheme yielded the highest sensitivity, specificity, accuracy, and G-mean of ca. 60%, suggesting that it performed better than the other two consensuses scoring schemes. Such observations are completely consistent with that made by Wang and Wang^43^. Nevertheless, little performance differences between the best scoring function and the best consensus scoring scheme, *viz. PMF* and rank-by-number, can be observed. This indicates that consensus scoring schemes do not always perform better than scoring functions in selecting the top-most poses. The average SVM-Pose still outperformed the 3 consensuses scoring schemes. Thus, it can be asserted that the SVM-based customized models are the best predictors to qualitatively and quantitatively accurately select the top-most poses especially in the case of ensemble docking for which multiple protein conformations are considered.

**Figure 11:**
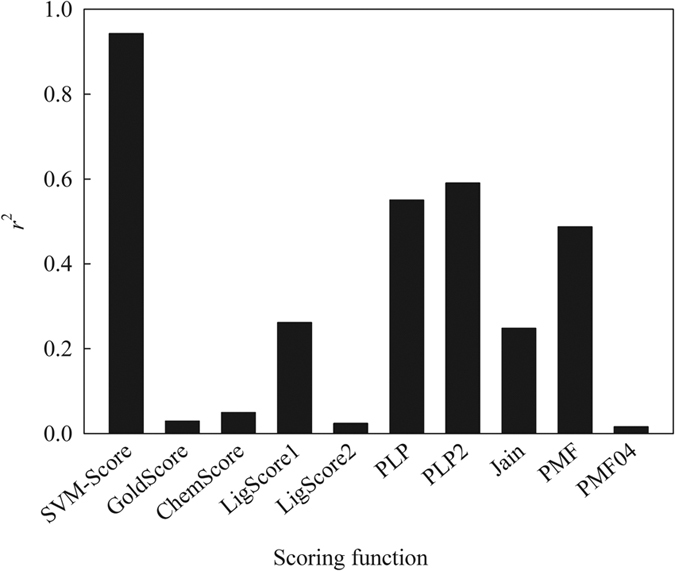
The correlation coefficient (*r*^2^) between predicted scores and p*K*_i_ values by SVM-Score and various scoring functions.

Of the scoring functions selected in this study, *PLP1, PLP2*, and *PMF* yielded the highest *r*^2^ values of ca. 0.60 between predicted scores and experimental p*K*_i_ values when considering all of samples used in this study, *viz*. training and test samples, as shown in Fig. 11. SVM-Score produced an even higher *r*^2^ of 0.97, suggesting that it outperformed the other scoring functions in correlating predicted scores and experimental p*K*_i_ values. Such substantial performance discrepancies indicate that SVM-Score is a nonlinear ML-based model *per se* as compared with the other linear scoring functions^60^. It has been demonstrated that the nonlinear SVM-based scheme can be more appropriate to render the relationship between independent variables, *viz*. descriptors in this study, and dependent variables, *viz*. p*K*_i_ values^89^. Furthermore, the descriptors adopted by SVM-Score appropriately augment the protein-ligand interactions, which are not otherwise selected by scoring functions.

When applied to selecting top-most poses, SVM-Score did not perform better than any other scoring functions qualitatively (Fig. 10) despite it outperformed the scoring functions in predicting binding affinities. For instance, SVM-Score yielded an MCC value of ca. −80%. Such performance discrepancies suggest that it is inappropriate to adopt a scoring function to select poses. As such, pose selections and binding affinity predictions should be carried out independently^114^,^115^.

**Figure 12:**
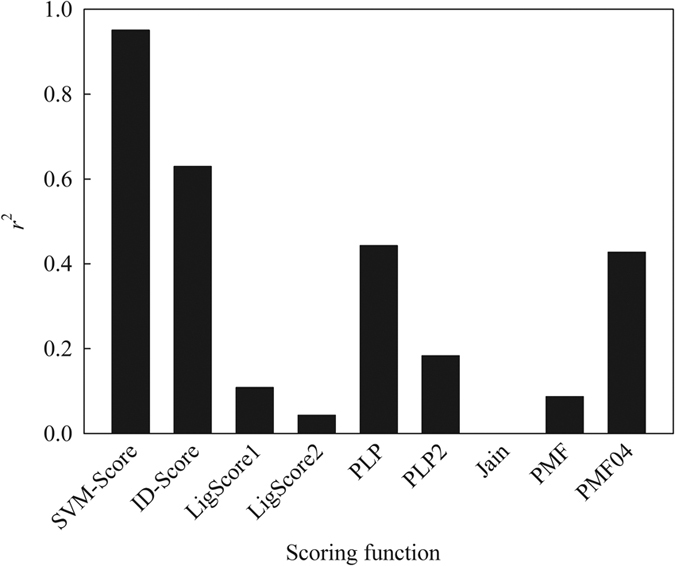
The correlation coefficient (*r*^2^) between predicted scores and p*K*_i_ values by SVM-Score, ID-Score, and various scoring functions based 6 common bound ligands.

Recently, Li *et al*. developed an empirical SVM-based *ID-Score* using various protein-ligand interactions^65^. Of all co-complex structures adopted by Li *et al*. for model development, 6 NMDAR crystal structures were also selected. As such, the binding affinities predicted by *ID-Score* were excerpted from their published data and subjected to further comparisons with SVM-Score and various scoring functions. Figure 12 shows the correlation coefficients between calculated scores and observed p*K*_i_ values. It can be observed that *ID-Score* yielded an *r*^2^ of 0.63, suggesting that the SVM-based *ID-Score* performed better than the linear scoring functions. SVM-Score, conversely, gave rise to an *r*^2^ of 0.95. The substantial difference in *r*^2^ (0.95 *vs*. 0.63) obviously indicates the superiority of SVM-Score. Thus, it can be concluded that the SVM-based scoring functions perform better than the linear scoring functions and a customized scoring function executes better than the general scoring functions. This is completely consistent with the fact that customized ML-based scoring functions perform better than general linear scoring functions^42^. Furthermore, SVM performs better than other ML-based schemes, namely ANN, GFA, and RF^56^,^116^.

## Conclusion

The GluN1 ligand-binding domain of *N*-methyl-D-aspartate receptor is a potential pharmacological target for various types of neurodegenerative illness. A novel combinatorial ensemble docking scheme was derived to predict the NMDA GluN1-ligand binding affinity using the customized SVM-based models to select the poses and to predict the binding affinities. The developed SVM-Pose models quantitatively predicted RMSD values well and qualitatively selected the top-most poses. The built SVM-Score accurately predicted the protein-ligand binding affinities and outperformed any scoring functions and consensus scoring functions. When mock tested by a group of novel molecules to mimic real world challenges, this novel docking scheme executed well. Thus, this novel customized combinatorial ensemble docking scheme is an accurate, predictive, and rapid tool for predicting the NMDAR GluN1-ligand binding affinity to facilitate and expedite the drug discovery and development of novel therapeutics to treat certain neurodegenerative illnesses.

## Additional Information

**How to cite this article**: Leong, M. K. *et al*. Prediction of *N*-Methyl-D-Aspartate Receptor GluN1-Ligand Binding Affinity by a Novel SVM-Pose/SVM-Score Combinatorial Ensemble Docking Scheme. *Sci. Rep.* **7**, 40053; doi: 10.1038/srep40053 (2017).

**Publisher's note:** Springer Nature remains neutral with regard to jurisdictional claims in published maps and institutional affiliations.

## Supplementary Material

Supplementary Information

Supplementary Table S6

Supplementary Table S7

## Figures and Tables

**Figure 1:**
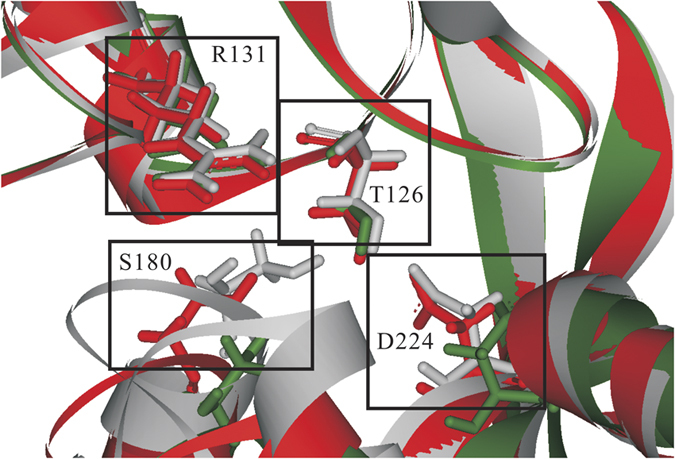
The superposition of proteins in various co-complex structures (PDB code: 1PB7, chain B of 1PBQ, chain A of 1Y1M), which are color-coded as gray, green, and red, respectively.

**Table 1:** Statistic evaluations and validation of SVM-Pose.

	SVM-Pose						
	1PB7	1PB8	1PB9	1PBQ	1Y1M	1Y1Z	1Y20
*r*^2^	0.98	0.99	0.97	0.94	0.98	0.98	0.93
Δ_Max_	0.00	0.09	0.42	0.14	0.49	0.49	0.57
MAE	0.00	0.02	0.19	0.02	0.17	0.37	0.14
*s*	0.00	0.02	0.12	0.03	0.16	0.18	0.21
RMSE	0.00	0.02	0.22	0.03	0.23	0.41	0.25
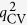	0.89	0.95	0.93	0.91	0.91	0.91	0.90
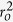	0.98	0.99	0.97	0.93	0.97	0.97	0.92
*k*	1.00	0.99	1.00	0.98	0.99	1.00	0.99
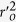	0.97	0.99	0.97	0.92	0.97	0.97	0.91
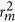	0.96	0.97	0.94	0.90	0.94	0.96	0.86
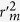	0.94	0.97	0.91	0.83	0.93	0.93	0.79
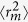	0.95	0.97	0.92	0.86	0.83	0.94	0.83
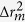	0.02	0.00	0.03	0.07	0.01	0.03	0.07
Eq. (1)	x	x	x	x	x	x	x
Eq. (2)	x	x	x	x	x	x	x
Eq. (3)	x	x	x	x	x	x	x
Eq. (4)	x	x	x	x	x	x	x
Eq. (5)	x	x	x	x	x	x	x
Eq. (6)	x	x	x	x	x	x	x

Statistic evaluations of SVM-Score models, namely correlation coefficient (*r*^2^), 10-fold cross-validation correlation coefficient (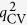), maximal absolute residual (Δ_Max_), mean absolute error (MAE), standard deviation (*s*), and RMSE as well as validation.

**Table 2:** Statistic evaluations and validation of SVM-Score.

	Training Set	Test Set
*n*	24	13
*r*^*2*^, *q*^*2*^	0.967	0.894
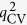	0.899	N/A†
Δ_Max_	0.667	0.729
MAE	0.244	0.391
*s*	0.170	0.202
RMSE	0.295	0.437
Eq. (1)	x	x
Eq. (2)	x	N/A†
Eq. (3)	x	N/A
Eq. (4)	x	x
Eq. (5)	x	x
Eq. (6)	x	x
Eq. (7)	N/A	x

^†^Not applicable.

Statistic evaluations of SVM-Score, namely correlation coefficients (*r*^2^ and *q*^2^), 10-fold cross-validation correlation coefficient (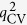), maximal absolute residual (Δ_Max_), mean absolute error (MAE), standard deviation (*s*), and RMSE as well as validation in the training set and test set.

**Table 3:** Selected descriptors for SVM-Score.

Descriptor	Description
*S*(*vdw_ext*)	External protein-ligand vdw contribution to *GoldScore* value
*S*(*vdw_int*)	Internal ligand vdw contribution to *GoldScore* value
HBD	Number of hydrogen-bond donor groups.
Atype_N_75	N in R–N–R or R–N–X
CIC1	Complementary information content index (neighborhood symmetry of 1-order)
CC	Count of the number of chiral centers (*R* or *S*) present in a molecule
JGI4	Mean topological charge index of order 4
CIC1	Complementary information content index (neighborhood symmetry of 1-order)
S_ssCH_2_	Sum descriptor for carbon with two single bonds.
Atype_N_75	N in R–N–R or R–N–X
HATS6u	Leverage-weighted autocorrelation of lag 6/unweighted

Descriptors selected as the input of SVM-Score and their descriptions.
