# Supplementary material for: Prediction of N-Methyl-D-Aspartate Receptor GluN1-Ligand Binding Affinity by a Novel SVM-Pose/SVM-Score Combinatorial Ensemble Docking Scheme
Source: Sci Rep. 2017 Jan 6;7:40053. doi: 10.1038/srep40053 (PMC5216401; doi:10.1038/srep40053)
Supplement: Supplementary Information [file srep40053-s1.pdf]

Prediction of *N*-Methyl-D-Aspartate Receptor GluN1-Ligand Binding Affinity by a Novel SVM-Pose/SVM-Score Combinatorial Ensemble Docking Scheme

Supplementary Information

Max K. Leong[1,2*], Ren-Guei Syu[1], Yi-Lung Ding[1], and Ching-Feng Weng[2*]

[1]Department of Chemistry, National Dong Hwa University, Shoufeng, Hualien 97401, Taiwan

[2]Department of Life Science and Institute of Biotechnology, National Dong Hwa University, Shoufeng, Hualien 97401, Taiwan

[*]Correspondence: Max Leong, leong@mail.ndhu.edu.tw; Ching-Feng Weng, cfweng@mail.ndhu.edu.tw

Table S1. Statistic parameters and their definitions.

Parameter	Definition†
r^2	$1-\sum_{i=1}^{n_{\mathrm{TR}}}(\hat{y}_i-y_i)^2 \Big/ \sum_{i=1}^{n_{\mathrm{TR}}}(y_i-\langle\hat{y}\rangle)^2$
q^2	$1-\sum_{i=1}^{n_{\mathrm{EXT}}}(\hat{y}_i-y_i)^2 \Big/ \sum_{i=1}^{n_{\mathrm{EXT}}}(y_i-\langle\hat{y}\rangle)^2$
q^2_{CV}	the correlation coefficient of 10-fold cross validation
Δ_i	$y_i-\hat{y}_i$
RMSE	$\left(\sum_{i=1}^{n}\Delta_i^2 \Big/ n\right)^{1/2}$
MAE	$\frac{1}{n}\sum_{i=1}^{n}\lvert\Delta_i\rvert$
r_m^2	$r^2\left(1-\sqrt{\lvert r^2-r_o^2\rvert}\right)$
$r_m'^2$	$r^2\left(1-\sqrt{\lvert r^2-r_o'^2\rvert}\right)$
$\langle r_m^2\rangle$	$\left(r_m^2+r_m'^2\right)\big/2$
q^2_{F1}	$1-\sum_{i=1}^{n_{\mathrm{EXT}}}(y_i-\hat{y}_i)^2 \Big/ \sum_{i=1}^{n_{\mathrm{EXT}}}(y_i-\langle y_{\mathrm{TR}}\rangle)^2$
q^2_{F2}	$1-\sum_{i=1}^{n_{\mathrm{EXT}}}(y_i-\hat{y}_i)^2 \Big/ \sum_{i=1}^{n_{\mathrm{EXT}}}(y_i-\langle y_{\mathrm{EXT}}\rangle)^2$
q^2_{F3}	$1-\left[\sum_{i=1}^{n_{\mathrm{EXT}}}(y_i-\hat{y}_i)^2 \big/ n_{\mathrm{EXT}}\right] \Big/ \left[\sum_{i=1}^{n_{\mathrm{TR}}}(y_i-\langle y_{\mathrm{TR}}\rangle)^2 \big/ n_{\mathrm{TR}}\right]$
CCC	$\dfrac{2\sum_{i=1}^{n_{\mathrm{EXT}}}(y_i-\langle y_{\mathrm{EXT}}\rangle)(\hat{y}_i-\langle\hat{y}_{\mathrm{EXT}}\rangle)}{\sum_{i=1}^{n_{\mathrm{EXT}}}(y_i-\langle y_{\mathrm{EXT}}\rangle)^2+(\hat{y}_i-\langle\hat{y}_{\mathrm{EXT}}\rangle)^2+n_{\mathrm{EXT}}(\langle y_{\mathrm{EXT}}\rangle-\langle\hat{y}_{\mathrm{EXT}}\rangle)^2}$

†where n, n_{TR}, n_{EXT}, y_i, $\hat{y}_i$, $\langle\hat{y}\rangle$, r_o^2, k, $r_o'^2$, represent the number of samples in the data set, the number of samples in the training set, the number of samples in the external set, the observed value, the predicted value, the average of predicted values, the correlation coefficient of the

regression line (predicted *vs*. observed values) through the origin, the slope of the regression line (predicted *vs*. observed values) through the origin, the correlation coefficient of the regression line (observed *vs*. predicted values) through the origin.

Table S2. Confusion matrix for qualitative predictive model.

		Observed +	Observed −
Predicted	+	true positive (TP)	false positive (FP)
	−	false negative (FN)	true negative (TN)

Table S3. The Cooper statistics and Kubat's G-mean calculated from the confusion matrix (Table S2)

Parameter	Definition[†]
Sensitivity	TP / (TP + FN)
Specificity	TN / (FP +TN)
Accuracy	(TP + TN) / *N*
Matthews Correlation Coefficient	$\frac{\mathrm{TP} \times \mathrm{TN} - \mathrm{FP} \times \mathrm{FN}}{\sqrt{(\mathrm{TP}+\mathrm{FP}) \times (\mathrm{TP}+\mathrm{FN}) \times (\mathrm{TN}+\mathrm{FP}) \times (\mathrm{TN}+\mathrm{FN})}}$
G-mean	$(\text{Sensitivity} \times \text{Specificity})^{1/2}$

[†] *N* = TP + TN + FP + FN.

Table S4. Optimal runtime parameters for the SVM models.

Model	SVM type	Kernel type	γ	Cost	ε
SVM-Pose[1PB7]	ε-SVR	RBF[†]	996	5.200×10^{-3}	2.500×10^{-3}
SVM-Pose[1PB8]	ε-SVR	RBF	320	7.813×10^{-4}	6.250×10^{-3}
SVM-Pose[1PB9]	ε-SVR	RBF	5000	6.250×10^{-6}	3.906×10^{-3}
SVM-Pose[1PBQ]	ε-SVR	RBF	5	3.125×10^{-3}	3.906×10^{-3}
SVM-Pose[1Y1M]	ε-SVR	RBF	4000	3.125×10^{-5}	7.813×10^{-3}
SVM-Pose[1Y1Z]	ε-SVR	RBF	312.5	1.563×10^{-4}	7.813×10^{-3}
SVM-Pose[1Y20]	ε-SVR	RBF	312.5	6.250×10^{-6}	3.906×10^{-3}
SVM-Score	ε-SVR	RBF	15551	2.572×10^{-4}	1.850×10^{-1}

[†]Radial basis function

Table S5. Descriptors selected by 7 SVM-Pose models.

Descriptor	SVM-Pose							Description
	1PB7	1PB8	1PB9	1PBQ	1Y1M	1Y1Z	1Y20	
S (hb_ext)	x	x	x	x	x		x	Protein-ligand H-bond contribution to *GoldScore* value
S (vdw_ext)		x	x	x	x	x		Protein-ligand vdw contribution to *GoldScore* value
S (vdw_int)	x	x		x	x	x	x	Internal ligand vdw contribution to *GoldScore* value
S (tor_int)	x	x		x		x	x	Internal ligand torsion-strain contribution to *GoldScore* value
ΔG		x	x	x	x		x	Free energy change (that occurs on ligand binding) contribution to *ChemScore* value
S (hbond)		x	x	x			x	Protein-ligand H-bond contribution to *ChemScore* value
S (lipo)	x	x	x	x	x		x	Protein-ligand lipophilic contribution to the *ChemScore* value
ΔE (clash)		x	x	x	x	x	x	Protein-ligand clash penalty to the *ChemScore*

								value
Δ*E* (int)	x	x		x	x		x	Internal ligand torsional strain penalty to the *ChemScore* value
LigScore1		x	x	x		x		
LigScore2	x		x	x	x	x	x	
PLP	x		x	x	x	x	x	Empirical scoring function
PLP2		x	x			x		
Jain	x	x	x	x	x	x		
LUDI2	x							
PMF		x	x	x		x	x	Knowledge-based scoring function
PMF04			x		x	x		

Table S8. The correlation coefficients (r) between selected descriptors and pK_i for specific chemotypes and the other molecules and between descriptors for all molecules enlisted in the SVM-Score development.

r	Molecules	
	Total (n = 37)	
HBD *vs*. *Gold.Chemscore.Hbond*	0.782	
	Bound ligand (n = 7)	The others
Atype_N_75 *vs*. HBA	0.881	0.318
	Total (n = 37)	
CIC1 *vs*. MR	0.800	
	Unbound ligands (n = 30)	The others
CC	−0.622	0.056
	Acetylenic aromatics (n = 5)	The others
JGI4 *vs*. pK_i	0.794	0.264
	Bound ligand (n = 7)	The others
S_ssCH_2 *vs*.pK_i	−0.786	−0.071
HATS6u *vs*. pK_i	−0.797	−0.294

Table S9. The observed pK_i values ([^{3}H]glycine) and predictive pK_i values ([^{3}H]MDL) for those molecules in the mock test.

Molecule	pK_i	
	Obs.	Pred.
Disodium;6,7-dinitroquinoxaline-2,3-diolate	6.41	6.02
7,8-Dichloro-1-ethyl-5H-[1,2,4]triazolo[4,3-a]quinoxalin-4-one	5.34	3.57
7,8-Dichloro-1-propyl-5H-[1,2,4]triazolo[4,3-a]quinoxalin-4-one	5.41	3.72
1-Butyl-7,8-dichloro-5H-[1,2,4]triazolo[4,3-a]quinoxalin-4-one	5.83	2.42
7,9-Dichloro-5H-[1,2,4]triazolo[4,3-a]quinoxalin-4-one	5.55	2.53
Nitro-5H-[1,2,4]triazolo[4,3-a]quinoxalin-4-one	5.13	3.68
7,8-Dinitro-5H-[1,2,4]triazolo[4,3-a]quinoxalin-4-one	5.29	3.18
7,8-Dichloro-5H-imidazo[1,2-a]quinoxalin-4-one	5.90	3.91
6,7-Dichloroquinoxaline-2,3-dione	6.68	4.99
5,7-Dichlorokynurenic acid	7.40	7.49

Figure legends

Figure S1. Molecular distribution for unbound (circles) and unbound samples (triangles) in the training set (filled) and test set (open) in the chemical space spanned by three principal components.

Figure S2. Histograms of (a) pK_i, (b) area, (c) molecular volume (V_m), and (d) molecular weight (MW) in density form for all molecules in the training set and test set.

Figure S3. Average pK_i versus the distribution of HBA for those bound ligands.

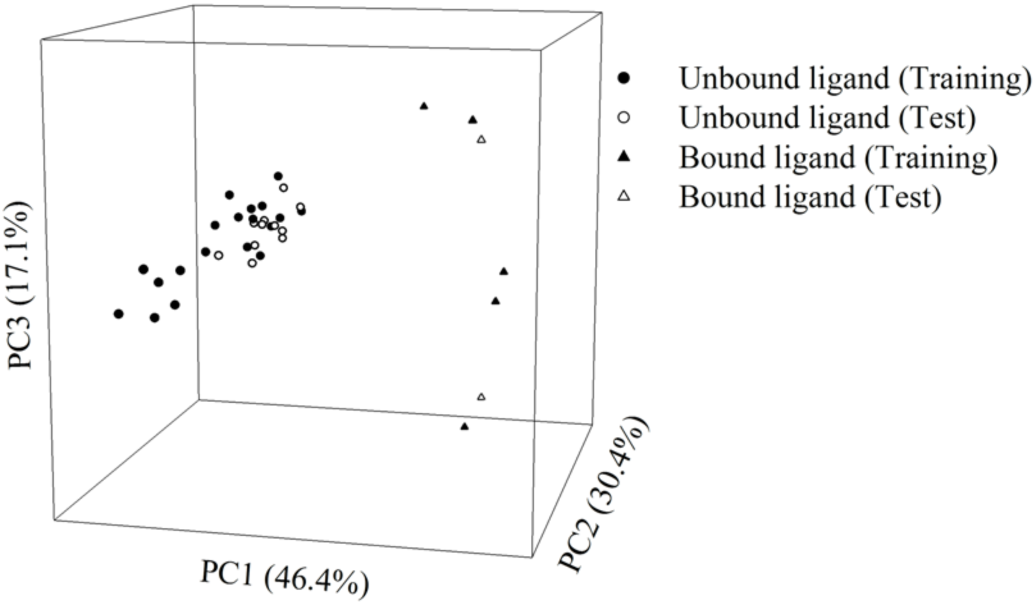

Figure S1. Molecular distribution for unbound (circles) and unbound samples (triangles) in the training set (filled) and test set (open) in the chemical space spanned by three principal components.

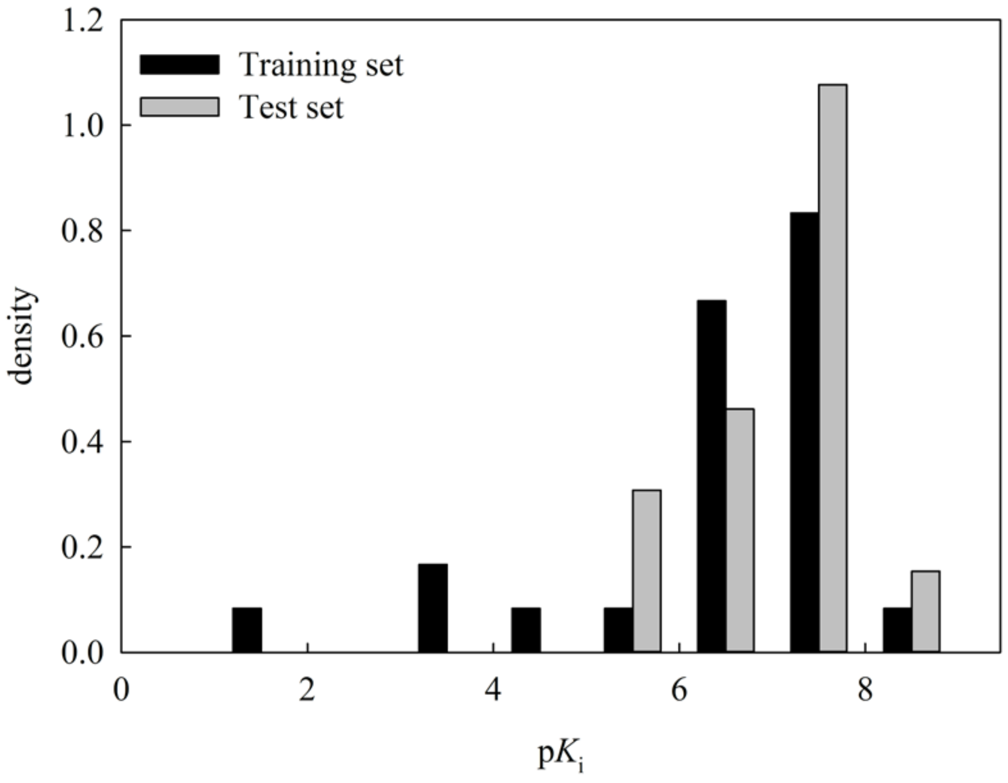

(a)

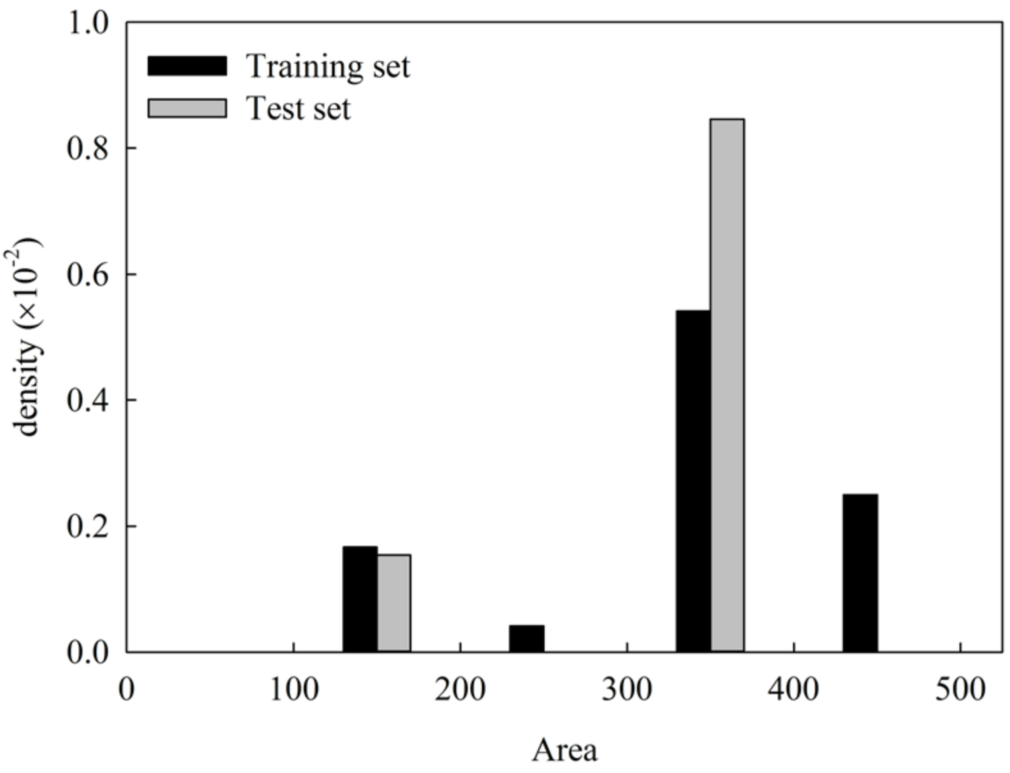
1.0
0.8
0.6
0.4
0.2
0.0
density (×10^-2)
Training set
Test set
0
100
200
300
400
500
Area

(b)

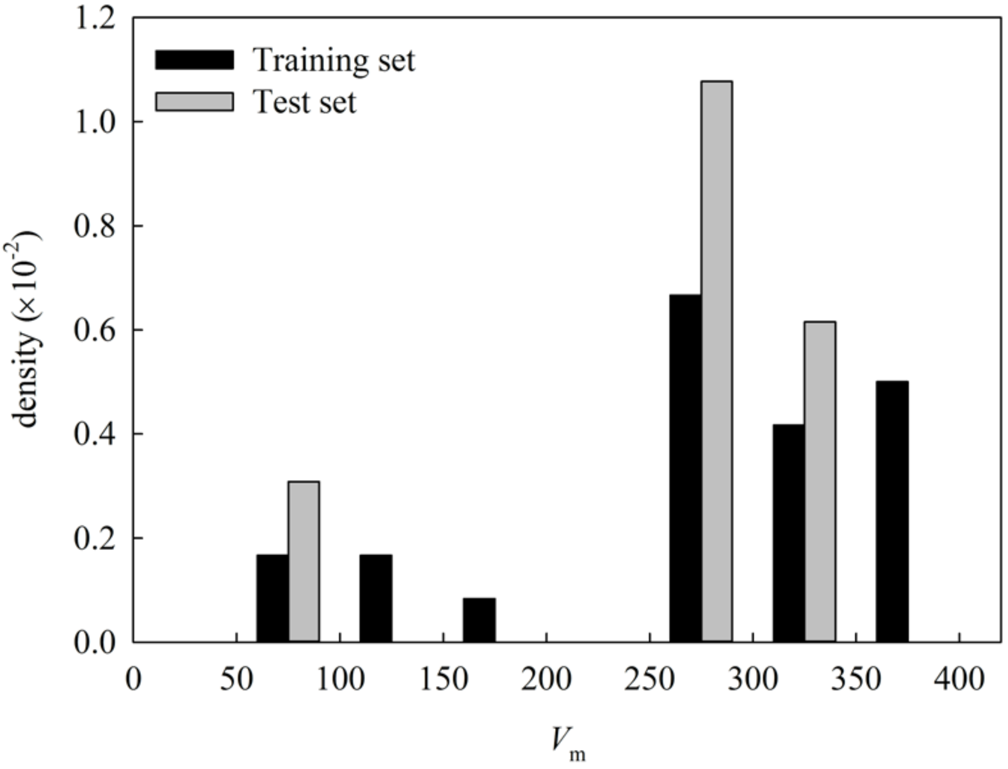
1.2
1.0
0.8
0.6
0.4
0.2
0.0
density (×10-2)
Training set
Test set
0
50
100
150
200
250
300
350
400
Vm

(c)

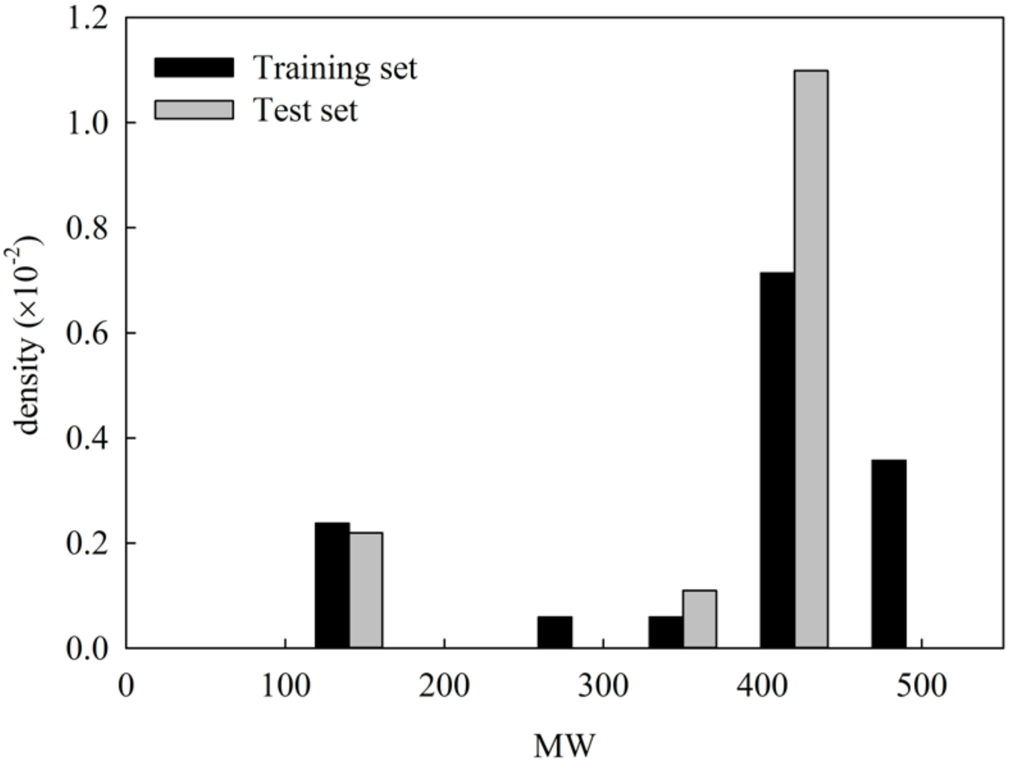

(d)

Figure S2. Histograms of (a) pK_i, (b) area, (c) V_m, and (d) MW in density form for all molecules in the training set and test set.

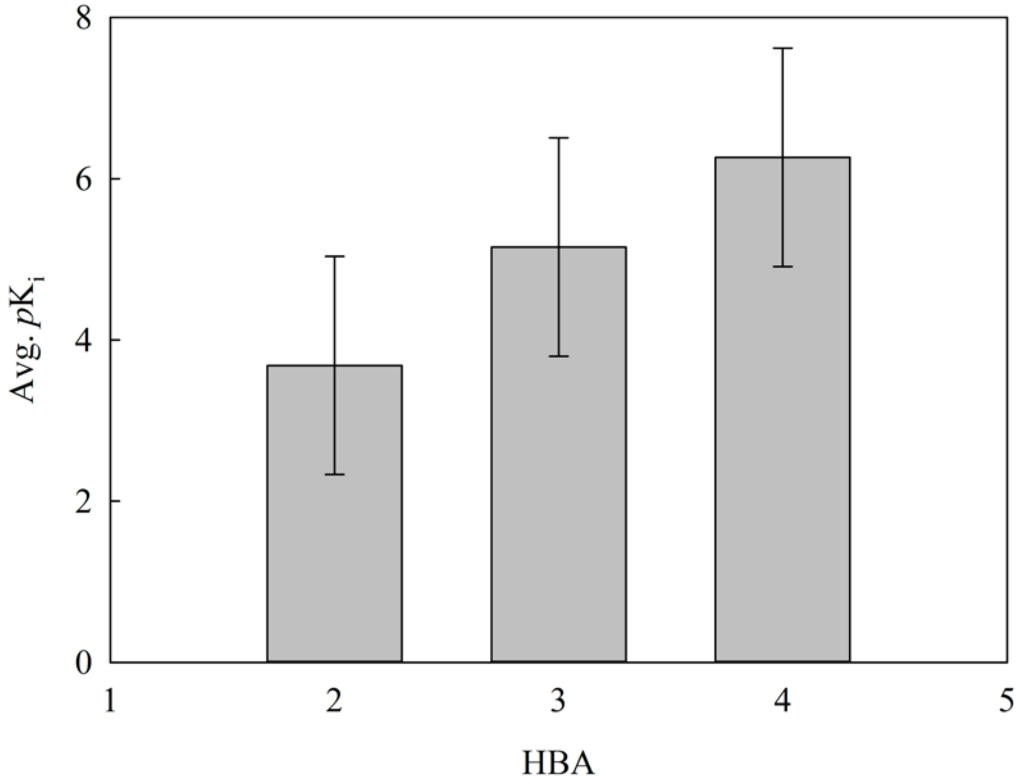

Figure S3. Average pK_i versus the distribution of HBA for those bound ligands.
